# Trefoil factor 2 secreted from damaged hepatocytes activates hepatic stellate cells to induce fibrogenesis

**DOI:** 10.1016/j.jbc.2021.100887

**Published:** 2021-06-17

**Authors:** Bichen Zhang, Kalina Lapenta, Qi Wang, Jin Hyun Nam, Dongjun Chung, Marie E. Robert, Michael H. Nathanson, Xiaoyong Yang

**Affiliations:** 1 Department of Cellular and Molecular Physiology, Yale University, New Haven, Connecticut, USA; 2 Program in Integrative Cell Signaling and Neurobiology of Metabolism, Department of Comparative Medicine, Yale University School of Medicine, New Haven, Connecticut, USA; 3 Department of Public Health Sciences, Medical University of South Carolina, Charleston, South Carolina, USA; 4 Department of Biomedical Informatics, College of Medicine, Ohio State University, Columbus, Ohio, USA; 5 Department of Pathology, Yale University School of Medicine, New Haven, Connecticut, USA; 6 Section of Digestive Diseases, Department of Internal Medicine, Yale University School of Medicine, New Haven, Connecticut, USA

**Keywords:** trefoil factor 2, OGT, PDGFRβ, hepatic stellate cell, intercellular signaling, liver fibrosis, αSMA, alpha-smooth muscle actin, AKT, protein kinase B, BSA, bovine serum albumin, CCl_4_, carbon tetrachloride, ECM, extracellular matrix, ERK, extracellular signal-regulated kinases, FBS, fetal bovine serum, GBSS/B, Gey′s balanced salt solution B, GI, gastrointestinal, HCC, hepatocellular carcinoma, HSC, hepatic stellate cell, OGT, O-GlcNAc transferase, OGT-LKO, liver-specific OGT KO, PFA, paraformaldehyde, TFF2, trefoil factor 2, PDGFRβ, platelet-derived growth factor receptor beta

## Abstract

Liver fibrosis is a common characteristic of chronic liver diseases. The activation of hepatic stellate cells (HSCs) plays a key role in fibrogenesis in response to liver injury, yet the mechanism by which damaged hepatocytes modulate the activation of HSCs is poorly understood. Our previous studies have established that liver-specific deletion of O-GlcNAc transferase (OGT)leads to hepatocyte necroptosis and spontaneous fibrosis. Here, we report that OGT-deficient hepatocytes secrete trefoil factor 2 (TFF2) that activates HSCs and contributes to the fibrogenic process. The expression and secretion of TFF2 are induced in OGT-deficient hepatocytes but not in WT hepatocytes. TFF2 activates the platelet-derived growth factor receptor beta signaling pathway that promotes the proliferation and migration of primary HSCs. TFF2 protein expression is elevated in mice with carbon tetrachloride-induced liver injury. These findings identify TFF2 as a novel factor that mediates intercellular signaling between hepatocytes and HSCs and suggest a role of the hepatic OGT–TFF2 axis in the process of fibrogenesis.

Chronic liver diseases affect more than 1.5 billion patients worldwide (1). Without proper intervention, chronic liver diseases progress to liver cancer, which is the global leading cause of cancer mortality. Liver fibrosis is the common pathological outcome regardless of the etiology of liver diseases and also a critical determinant for the development of hepatocellular carcinoma (HCC) (2). Liver fibrosis is reversible if the primary cause of the liver injury is removed or limited. However, no Food and Drug Administration–approved drug is available for liver fibrosis *per se*. Understanding the cellular and molecular mechanisms underlying the hepatic fibrogenesis is critical for the identification of novel drug targets and the development of therapies.

Liver fibrosis is defined by excessive deposition of the extracellular matrix (ECM) in the tissue. The hepatic stellate cell (HSC) is the major cell type that contributes to liver fibrogenesis. Activated HSCs transdifferentiate from quiescent, fat-storing cells into proliferative myofibroblasts, which is marked by the loss of lipid droplets and the expression of alpha-smooth muscle actin (αSMA) (3). Activated HSCs show increased proliferation, chemotaxis, and contractility, as well as synthesis and secretion of the ECM. Lineage-tracing experiments in several animal models of liver fibrosis have shown that HSCs are the primary source of ECM production (4). In contrast, HSC apoptosis and senescence promote the resolution of liver fibrosis (5, 6). Therefore, investigating the mechanisms for the activation of HSCs may hold the key to treat liver fibrosis.

The crosstalk between hepatocytes and nonparenchymal cells is an emerging paradigm in the pathogenesis of liver diseases. Located along the sinusoids of the liver, the spread and elongated morphology of HSCs allows them to make contact with both hepatocytes and endothelial cells (7). However, our current knowledge of how paracrine factors released from damaged hepatocytes affect HSCs remains very limited, and the contribution of the hepatocyte–HSC crosstalk to liver fibrogenesis is unknown.

O-GlcNAc modification post-translationally modifies cytoplasmic, nuclear, and mitochondrial proteins (8, 9, 10, 11). Although over 1000 targets have been identified for O-GlcNAc modification, only one pair of enzymes is responsible for the attachment and removal of this modification. O-GlcNAc transferase (OGT) adds O-GlcNAc to serine or threonine residues on proteins, and O-GlcNAcase removes this sugar moiety. Maintaining optimal O-GlcNAcylation levels in the liver has been found to be important for liver metabolism and tissue homeostasis (12, 13, 14, 15, 16, 17, 18, 19, 20). Previously, we have discovered that deletion of OGT in hepatocytes leads to necroptotic cell death (21). Mice lacking OGT in hepatocytes spontaneously develop liver fibrosis, which could only be partially attributed to hepatocyte necroptosis. Here, we report that the HSCs from liver-specific OGT KO (OGT-LKO) mice are activated by a novel secreted factor trefoil factor 2 (TFF2). TFF2 activates PDGFRβ signaling and promotes the proliferation and migration of HSCs. TFF2 is also increased in mice with liver injury, which indicates a potential role in the pathogenesis of liver diseases.

## Results

### HSCs are activated in the OGT-LKO mouse model of liver fibrosis

**Figure 1:**
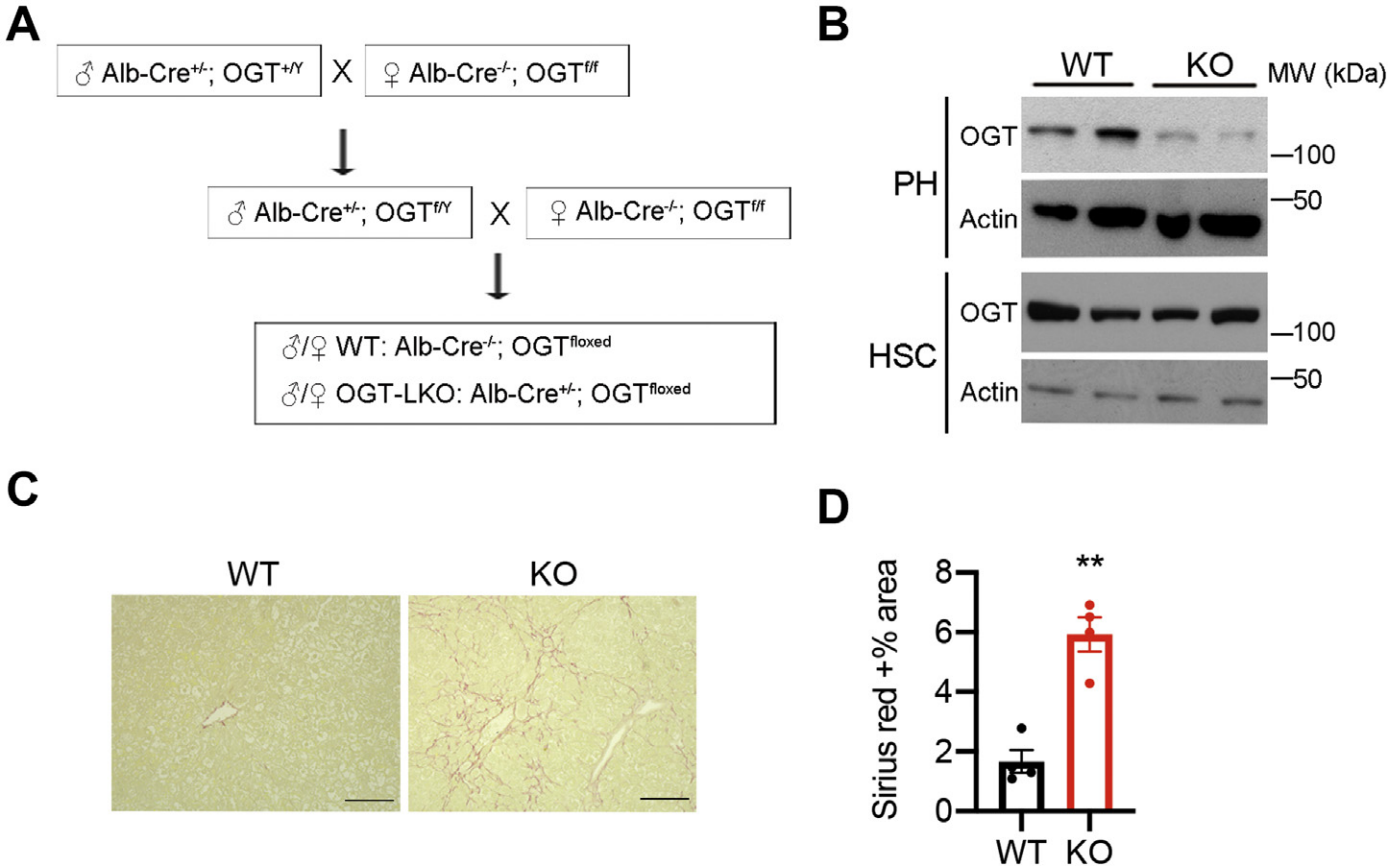
**Liver-specific OGT knockout (OGT-LKO) mice show fast-developed liver fibrosis.***A*, the breeding strategy of OGT-LKO mice. *B*, Western blotting of proteins extracted from primary hepatocytes (PH) and primary hepatic stellate cells (HSCs). All primary cells were isolated from 4-week-old mice. *C*, Sirius Red stains of liver sections from 8-week-old WT and OGT-LKO mice. The scale bar represents 100 μm. *D*, quantification of Sirius Red–positive area (n = 4). Data are shown as the mean ± SEM. ∗∗*p* < 0.01 by unpaired Student’s *t* test. OGT, O-GlcNAc transferase.

**Figure 2:**
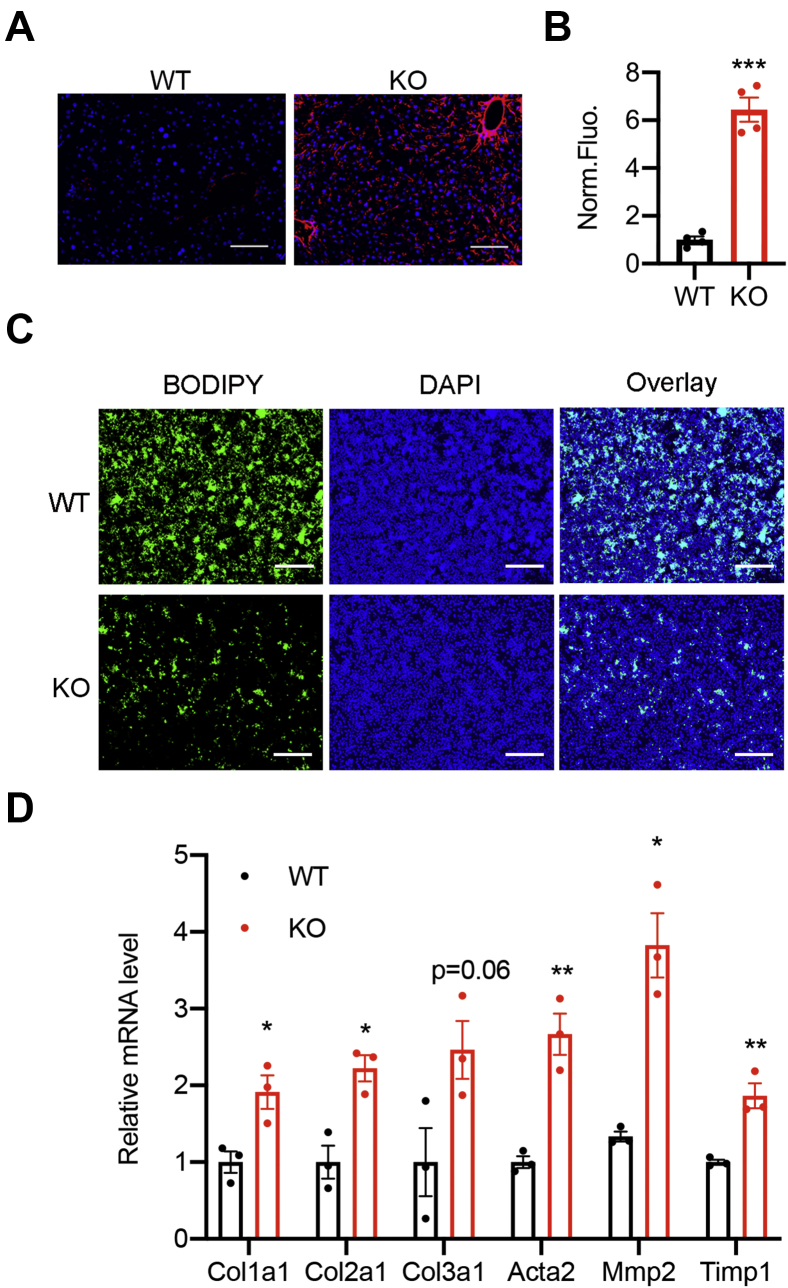
**Primary HSCs are activated in OGT-LKO mice.***A* and *B*, immunofluorescence images of liver sections stained with the antibody against alpha-smooth muscle actin (αSMA, *red*) and 4′,6-diamidino-2-phenylindole (*blue*). Fluorescence intensity was quantified with ImageJ (n = 4). The scale bar represents 100 μm. *C*, BODIPY stains of primary HSCs isolated from 4-week-old WT and OGT-LKO mice. 4′,6-diamidino-2-phenylindole was used to stain the nucleus. The scale bar represents 500 μm. *D*, mRNA expression of fibrogenic genes in primary HSCs isolated from 4-week-old WT and OGT-LKO mice, n = 3. Data are shown as the mean ± SEM. ∗*p* < 0.05; ∗∗*p* < 0.01; ∗∗∗*p* < 0.001 by unpaired Student’s *t* test. HSCs, hepatic stellate cells; OGT, O-GlcNAc transferase; OGT-LKO, liver-specific OGT KO.

We generated OGT-LKO mice by crossing *Albumin-Cre*; *Ogt*^*F/Y*^ mice with *Ogt*^*F/F*^ mice to specifically delete OGT in hepatocytes (Fig. 1, *A* and *B*). We reported that OGT-deficient hepatocytes undergo excessive necroptosis that contributes to liver fibrogenesis in these mice (21). We observed extensive Sirius Red–positive stains in 8-week-old OGT-LKO mouse liver sections, which confirmed the spontaneous development of liver fibrosis in these mice (Fig. 1, *C* and *D*). The expression of αSMA is a hallmark of the transdifferentiation of quiescent HSCs to the activated fibroblasts. Immunofluorescence staining of liver sections showed that αSMA is highly expressed in livers of 4-week-old OGT-LKO mice but not WT mice (Fig. 2, *A* and *B*). To further characterize HSCs from OGT-LKO mice, we isolated primary HSCs from WT and OGT-LKO livers. Quiescent HSCs are the major sites in the liver that stores retinoids and their metabolites in the form of lipid droplets (22). Multiple lipid droplets were presented in freshly isolated HSCs from WT livers as demonstrated by the BODIPY staining (Fig. 2*C*). In contrast, primary HSCs from OGT-LKO mice showed a significant decrease in the lipid content, indicating the transdifferentiation of HSCs into fibroblasts (Fig. 2*C*) (23). In agreement with these findings, mRNA levels of a battery of fibrogenic genes were elevated in primary HSCs from OGT-LKO mice (Fig. 2*D*). These results demonstrate that OGT deletion in hepatocytes promotes the transdifferentiation and activation of HSCs.

### OGT-deficient hepatocytes activate HSCs through a paracrine mechanism

**Figure 3:**
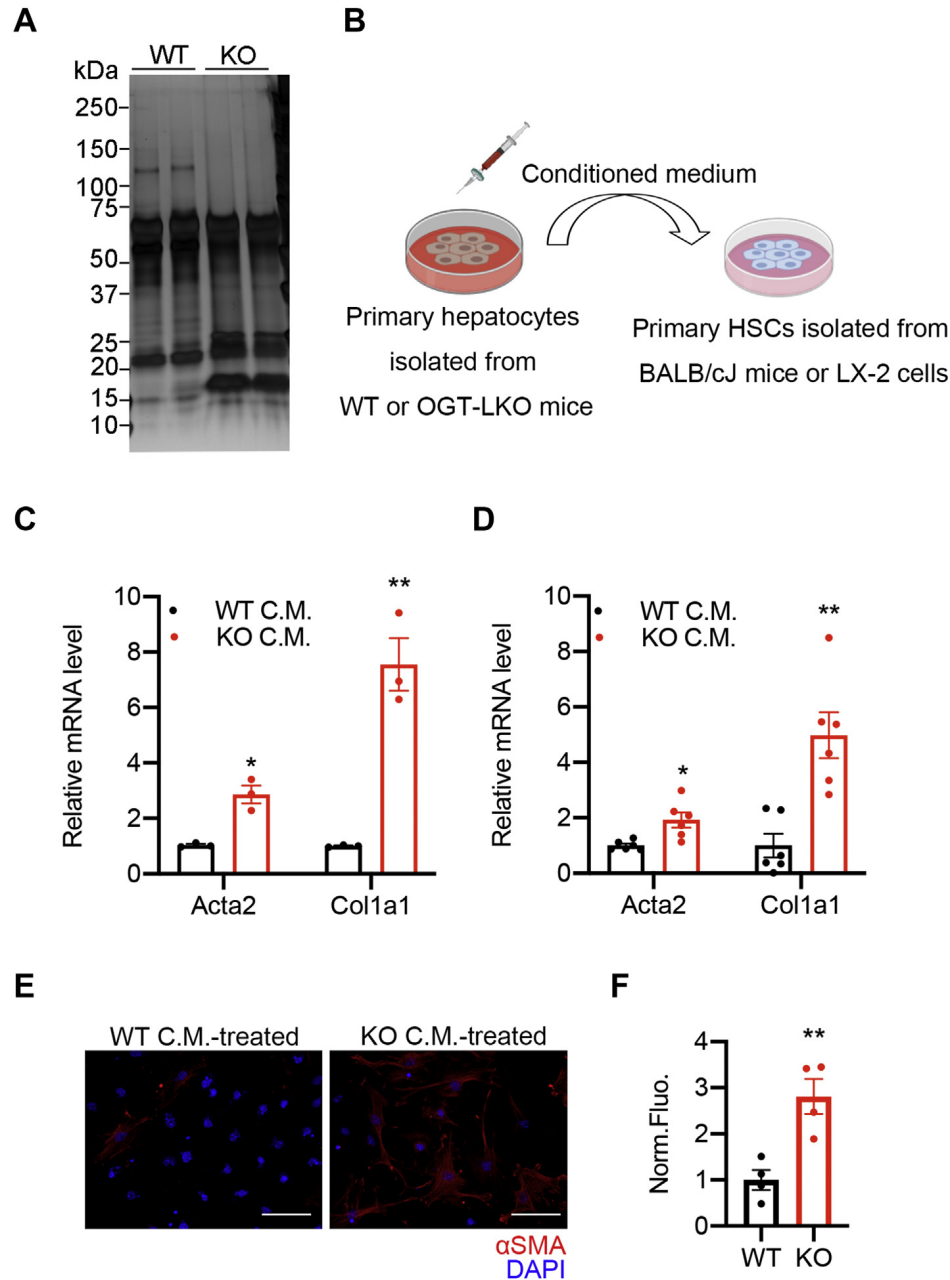
**OGT-deficient hepatocytes secrete proteins that activate HSCs.***A*, silver staining of proteins extracted from WT and OGT-LKO primary hepatocyte–conditioned medium (CM). Primary hepatocytes were cultured in FBS-free medium for 16 h. The conditioned medium was centrifuged using the Amicon filter system with a molecular weight cutoff of 3 kDa. Secreted proteins were precipitated with trichloroacetic acid and solubilized with urea. *B*, schematic view of the experimental design. *C*, mRNA expression of *Acta2* and *Col1a1* in primary HSCs treated with WT or KO hepatocyte-conditioned medium. *D*, mRNA expression of *Acta2* and *Col1a1* in LX-2 cells treated with WT or KO hepatocyte-conditioned medium. *E* and *F*, immunofluorescence images of HSCs treated with WT or KO hepatocyte-conditioned medium. Cells were stained with the antibody against αSMA (*red*) and 4′,6-diamidino-2-phenylindole (*blue*). Fluorescence intensity was quantified with ImageJ (n = 4). The scale bar represents 100 μm. Data are shown as the mean ± SEM. ∗*p* < 0.05; ∗∗*p* < 0.01 by unpaired Student’s *t* test. FBS, fetal bovine serum; HSCs, hepatic stellate cells; OGT, O-GlcNAc transferase; OGT-LKO, liver-specific OGT KO.

To understand how the deletion of OGT in hepatocytes affects the activation of neighboring HSCs, we cultured primary hepatocytes in fetal bovine serum (FBS)-free media overnight and harvested secreted proteins in the conditioned medium. The silver staining of these secreted proteins showed different patterns between WT and OGT-LKO primary hepatocytes (Fig. 3*A*). We therefore hypothesized that changes in these secreted proteins would affect the status of HSCs. To test this hypothesis, we treated primary HSCs and LX-2 cells (an immortalized human stellate cell line) with the conditioned medium harvested from WT and OGT-deficient hepatocytes for 16 h (Fig. 3*B*) (24). The expression of key fibrogenic genes, *Acta2* and *Col1a1*, was significantly increased in both primary HSCs and LX-2 cells treated with the OGT-deficient hepatocyte-conditioned medium compared with those treated with the WT hepatocyte-conditioned medium (Fig. 3, *C* and *D*). The immunofluorescence analysis confirmed that the αSMA protein level was increased in primary HSCs treated with the OGT-deficient hepatocyte-conditioned medium (Fig. 3, *E* and *F*). These results demonstrate that proteins secreted from OGT-deficient hepatocytes can induce the activation of HSCs.

### OGT-deficient hepatocytes show elevated expression and secretion of TFF2

**Figure 4:**
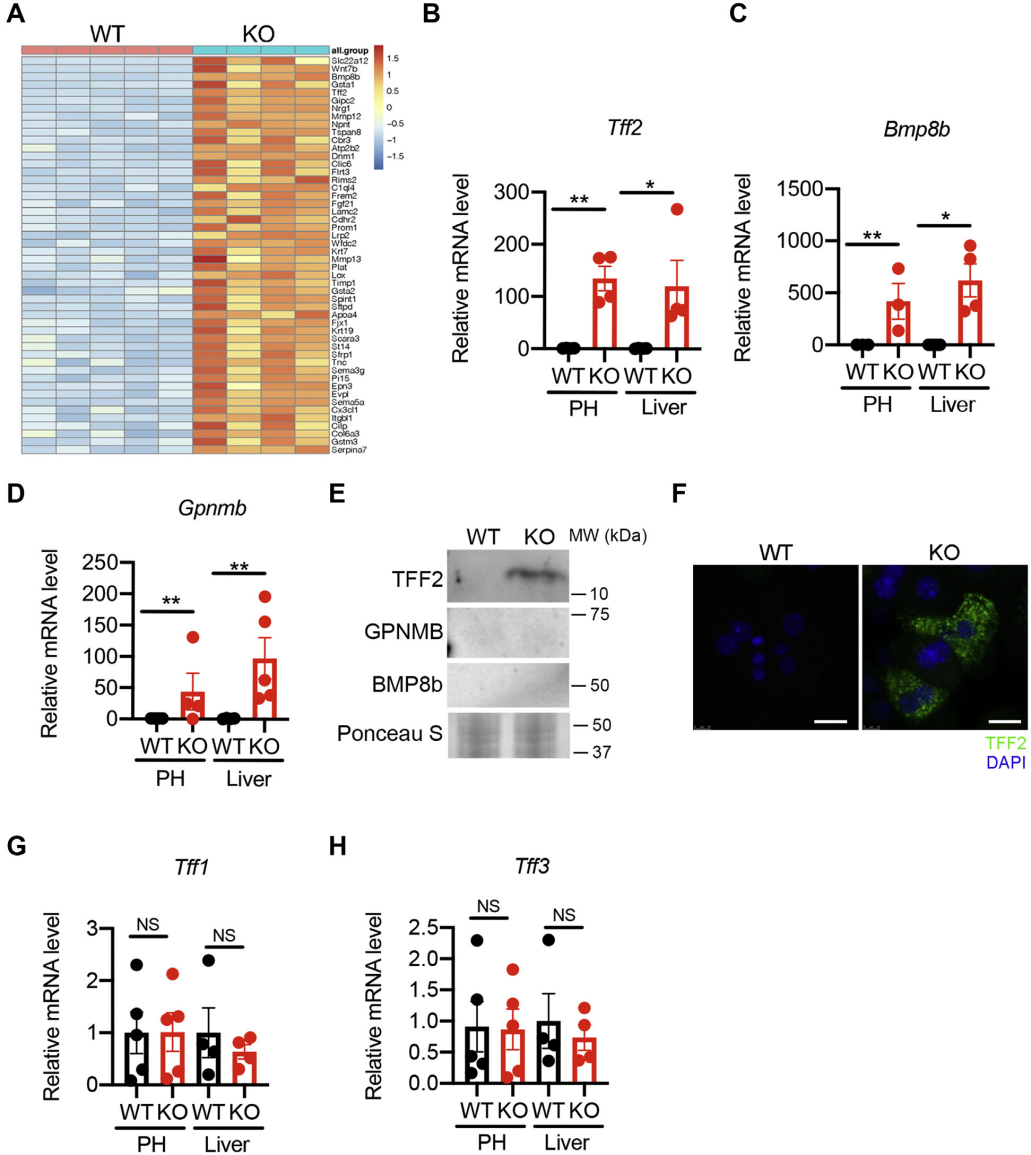
**The expression and secretion of trefoil factor 2 (TFF2) is increased in OGT-deficient hepatocytes.***A*, the expression of genes that encode secreted proteins in WT and OGT-LKO livers. Top 50 genes are shown based on the ranks of fold change. *B*–*D*, mRNA expression of *Tff2* (*B*), *Bmp8b* (*C*), and *Gpnmb* (*D*) in primary hepatocytes (PH) and liver lysates. Cell and liver lysates were isolated from 4-week-old WT and OGT-LKO mice. n = 3 to 5. *E*, Western blotting of proteins extracted from WT and KO hepatocyte-conditioned medium. *F*, immunofluorescence imaging of primary hepatocytes with the antibody against TFF2 (*green*) and 4′,6-diamidino-2-phenylindole (*blue*). The scale bar represents 25 μm. *G* and *H*, mRNA expression of *Tff1* and *Tff3* in primary hepatocytes and liver lysates. Cell and liver lysates were isolated from 4-week-old WT and OGT-LKO mice. n = 4 to 5. Data are shown as the mean ± SEM. ∗*p* < 0.05; ∗∗*p* < 0.01 by unpaired Student’s *t* test. HSCs, hepatic stellate cells; OGT, O-GlcNAc transferase; OGT-LKO, liver-specific OGT KO.

To identify potential candidates in the conditioned medium that mediate the effect on HSC activation, we re-examined the RNA-sequencing data of the OGT-LKO livers (21). A variety of genes that encode secreted proteins were differently expressed in WT and OGT-LKO livers (Fig. 4*A*). To identify secreted factors that may mediate the crosstalk between hepatocytes and HSCs, we chose *Tff2*, *Bmp8b*, and *Gpnmb* for the initial validation. These factors were chosen because they were upregulated in OGT-LKO livers and highly relevant to the pathogenesis of chronic liver diseases based on the analysis of the Gene Expression Omnibus database. We found that the mRNA levels of all three candidates were increased in OGT-LKO primary hepatocytes and livers, confirming the cell-autonomous upregulation of these genes in OGT-deficient hepatocytes (Fig. 4, *B*–*D*). To test whether the increase in these gene transcripts results in elevated secretion of the corresponding proteins, we analyzed the protein extracts from the WT and OGT-LKO hepatocyte-conditioned medium. We found that TFF2 but not BMP8b and GPNMB was present in the OGT-LKO hepatocyte-conditioned medium (Fig. 4*E*). Immunofluorescence staining of primary hepatocytes further demonstrated the expression of TFF2 protein in OGT-LKO but not WT hepatocytes (Fig. 4*F*). There are three members in the TFF family: *Tff1*, *Tff2*, and *Tff3* (25). We found no significant changes in *Tff1* and *Tff3* mRNA levels in OGT-LKO hepatocytes, indicating the specificity of increased *Tff2* transcription in response to *Ogt* deletion (Fig. 4, *G* and *H*). In summary, these results show that OGT deficiency in hepatocytes induces the expression and secretion of TFF2.

### TFF2 promotes the proliferation and migration of HSCs

**Figure 5:**
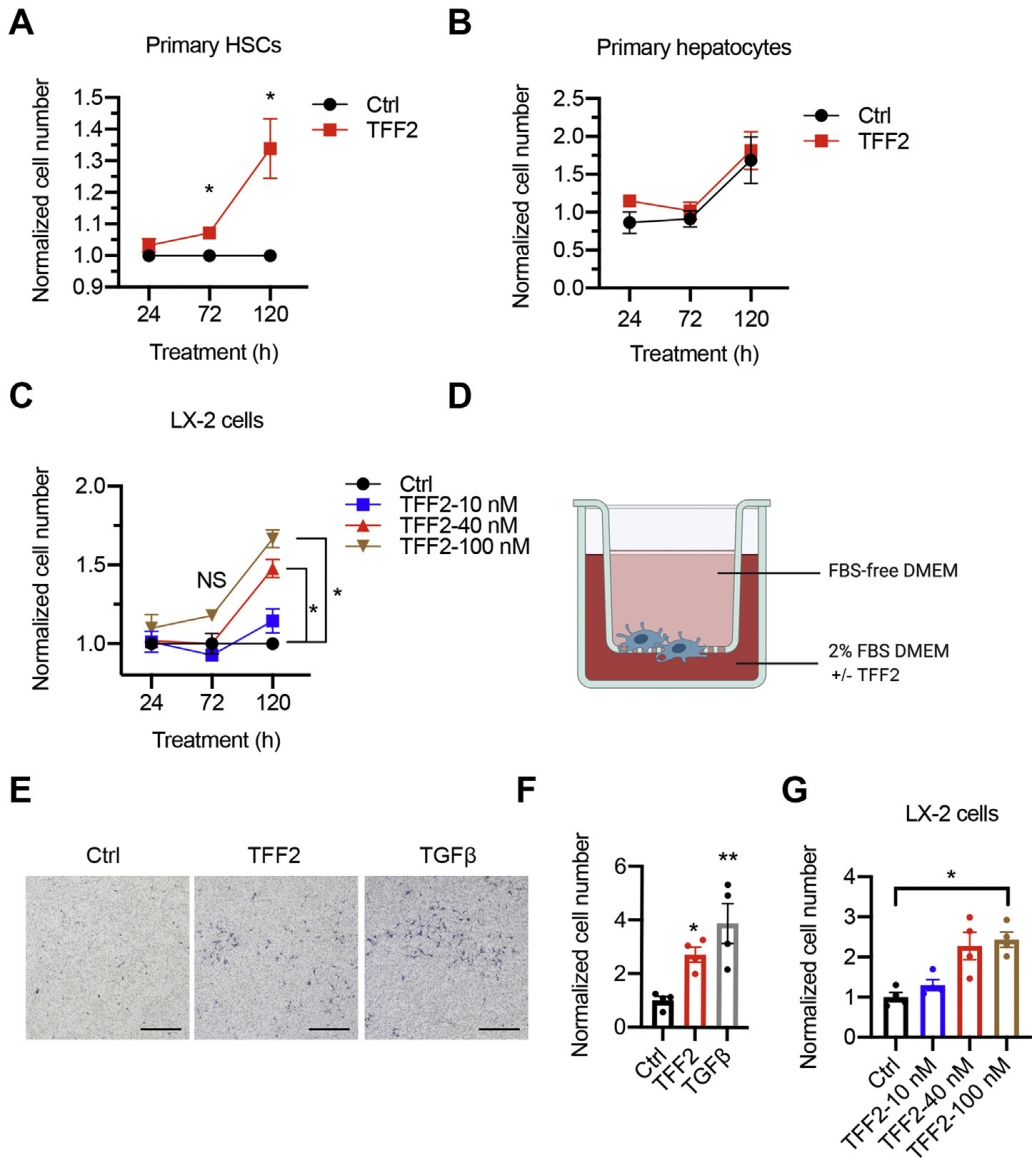
**TFF2 promotes the proliferation and migration of HSCs.***A* and *B*, cell proliferation analysis of primary HSCs (*A*) and primary hepatocyte (*B*) determined by MTT assay. Cells were treated with 40 nM TFF2 for 5 days. Cells in the control group were treated with 0.1% bovine serum albumin PBS. Fresh medium was replaced every 48 h. *C*, cell proliferation analysis of LX-2 cells treated with indicated concentration of TFF2. *D*, schematic view of the modified Boyden chamber assay. *E* and *F*, crystal violet staining and quantification of primary HSCs migrated to the lower membrane after 4 h treatment. The scale bar represents 500 μm. *G*, quantification of migrated LX-2 cells treated with indicated concentration of TFF2. Cells were isolated from 4-week-old mice, n = 3 to 4. Data are shown as the mean ± SEM. ∗*p* < 0.05; ∗∗*p* < 0.01 by unpaired Student’s *t* test and one-way ANOVA followed by Tukey-adjusted multiple comparisons. HSCs, hepatic stellate cells; TFF2, trefoil factor 2.

To investigate whether secreted TFF2 might mediate the crosstalk between hepatocytes and HSCs, we treated freshly isolated primary HSCs with recombinant TFF2. Activated HSCs demonstrate the increased capability to proliferate and migrate (3). We found that TFF2 treatment promoted the proliferation of primary HSCs compared with the control group (Fig. 5*A*). To determine whether this effect is specific to HSCs, we also treated primary hepatocytes with recombinant TFF2 and observed no difference in the cell number compared with the control group (Fig. 5*B*). We further tested the effect of TFF2 on LX-2 cells and found that TFF2 increased the proliferation of these human stellate cells in a dose-dependent manner (Fig. 5*C*). We then examined whether TFF2 can affect HSC migration with a modified Boyden chamber (Fig. 5*D*). Transforming growth factor beta was used as a positive control in the assay. With TFF2 treatment, more cells were migrated to the lower membrane in the Boyden chamber (Fig. 5, *E* and *F*). Similarly, TFF2 facilitated the migration of LX-2 cells in a dose-dependent manner (Fig. 5*G*). Taken together, these results indicate that secreted TFF2 promotes the proliferation and migration of HSCs.

### TFF2 treatment induces PDGFR phosphorylation and activation of downstream signaling

**Figure 6:**
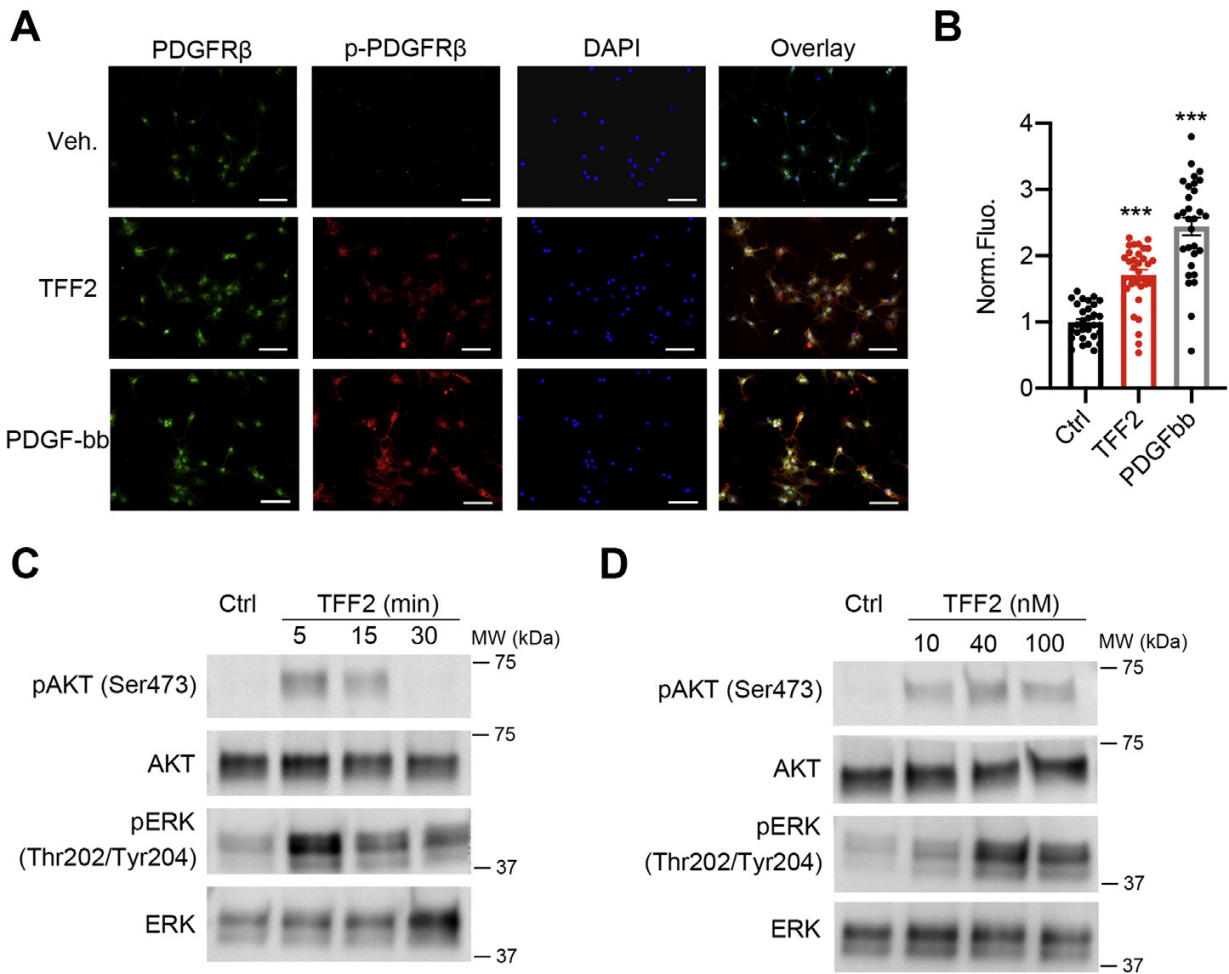
**TFF2 promotes the activation of PDGFRβ signaling.***A* and *B*, immunofluorescence imaging of HSCs with recombinant TFF2 treatment. PDGF-bb was used as a positive control. The scale bar represents 25 μm. *B*, quantification of the immunofluorescence images. The fluorescence intensity of p-PDGFRβ/total PDGFRβ was quantified and then normalized to the control group. n = 30. *C*, Western blots of primary HSCs treated with 40 nM TFF2 for 5, 15, and 30 min. *D*, Western blots of primary HSCs treated with different doses of TFF2 for 5 min. Cells were isolated from 4-week-old mice. Data are shown as the mean ± SEM. ∗∗∗*p* < 0.001 by one-way ANOVA followed by Tukey-adjusted multiple comparisons. HSCs, hepatic stellate cells; TFF2, trefoil factor 2.

We next sought to dissect the signaling pathways mediated by TFF2 in the activation of HSCs. Previous research demonstrated that the activation of the PDGFRβ signaling pathway governs the proliferation and migration of HSCs (26). We examined whether TFF2 can activate the PDGFRβ signaling pathway in primary HSCs. Immunofluorescence staining of cells treated with recombinant TFF2 or PDGF-bb for 10 min showed a marked increase in phospho-PDGFRβ but not total PDGFRβ protein levels, which indicated the activation of the PDGFRβ signaling (Fig. 6, *A* and *B*). Protein kinase B (AKT) and extracellular signal-regulated kinases (ERK) signaling pathways are downstream of PDGFRβ activation. We found that TFF2 treatment increased the phosphorylation of AKT and ERK in primary HSCs in a time- and dose-dependent manner (Fig. 6, *C* and *D*). These results together indicate that TFF2 exerts its effect on HSC proliferation and migration at least partially through the PDGFRβ signaling pathway.

### Increased expression of TFF2 in mice with carbon tetrachloride-induced liver injury

**Figure 7:**
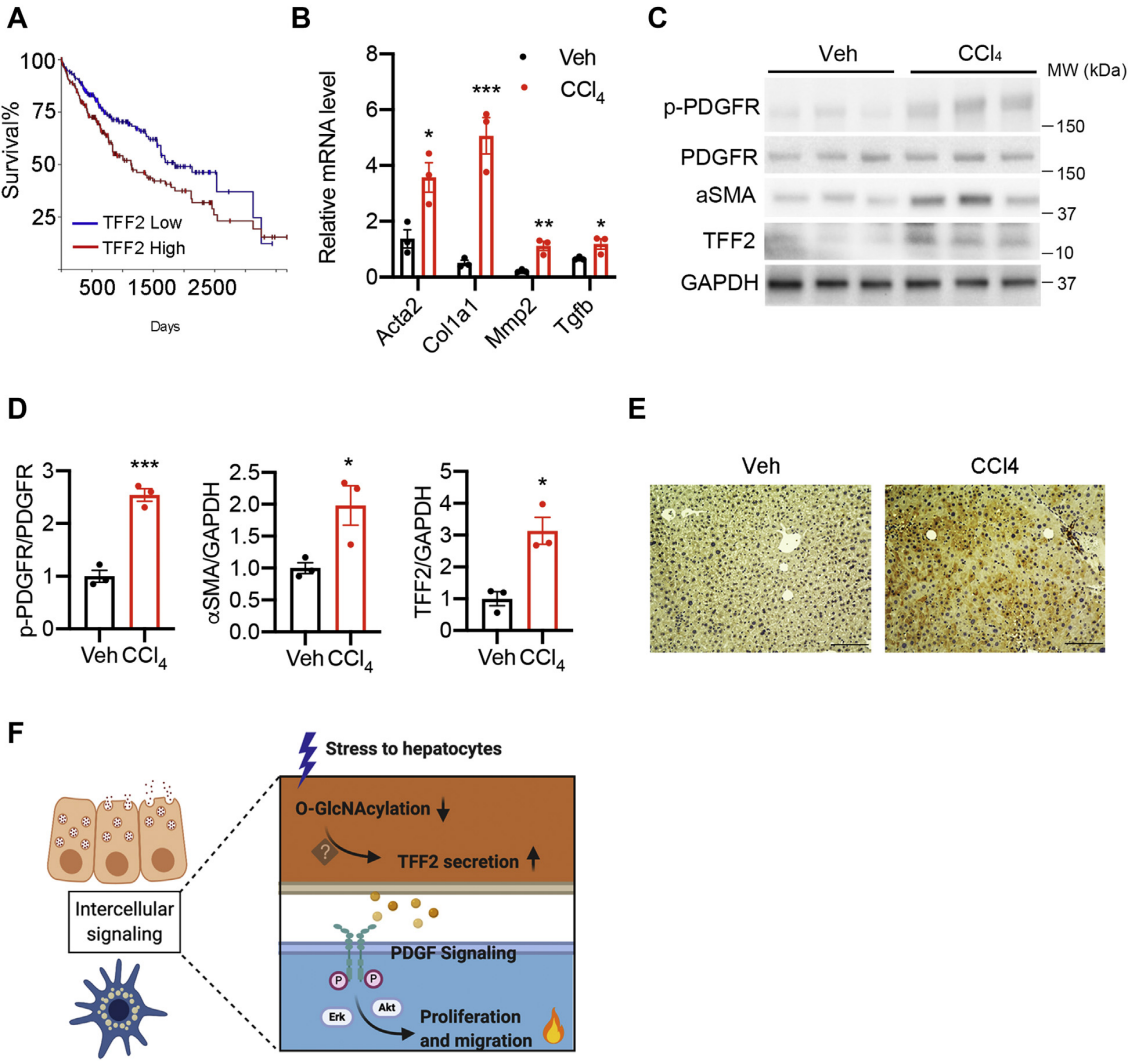
**Increased expression of TFF2 in mice with CCl**_**4**_**-induced liver injury.***A*, Kaplan–Meier plot for patients with hepatocellular carcinoma. Data source: the cancer genome atlas. Visualization: https://xenabrowser.net/. *B*, mRNA expression of fibrogenic genes in mice with 1-week vehicle or CCl_4_ injection. *C*, Western blotting of liver lysates from mice treated with vehicle or CCl_4_ for 1 week. *D*, quantifications of Western blot images. *E*, immunohistochemistry stains of TFF2 in mice treated with vehicle or CCl_4_ for 3 weeks. The scale bar represents 100 μm. *F*, working model of the intercellular signaling between OGT-deficient hepatocytes and HSCs. Data are shown as the mean ± SEM. ∗*p* < 0.05; ∗∗*p* < 0.01; ∗∗∗*p* < 0.001 by unpaired Student’s *t* test. HSCs, hepatic stellate cells; OGT, O-GlcNAc transferase; TFF2, trefoil factor 2.

TFF2 has been shown to be a poor prognostic marker for gastric cancer (27). We probed the cancer genome atlas database and found a similar trend in HCC: patients with higher expression of *Tff2* showed a lower survival rate (Fig. 7*A*). Because liver fibrosis is highly correlated to the development of HCC, we ask whether TFF2 is involved in the progression of liver diseases. To test the potential role of TFF2 in the mouse model of liver injury, we induced an acute liver injury by injecting carbon tetrachloride (CCl_4_) to C57BL/6J mice for 1 week (28). The fibrogenic program was initiated in CCl_4_-treated mice as demonstrated by increased mRNA expression of major fibrogenic genes (Fig. 7*B*). We found that TFF2 protein expression was increased in CCl_4_-treated mice along with the increased levels of αSMA and phospho-PDGFRβ proteins (Fig. 7, *C* and *D*). Immunohistochemistry staining of livers from mice injected with CCl_4_ for 3 weeks also showed higher expression of TFF2 than that from vehicle-treated mice (Fig. 7*E*). These results indicate a strong association between TFF2 expression and the early stage of liver fibrosis in these mice.

## Discussion

Hepatocytes show a remarkable capability to synthesize and secrete proteins that mediate intercellular and interorgan crosstalk. Changes in the intercellular crosstalk are observed in various liver pathological conditions, which indicate the critical role of this signaling network in the pathogenesis of liver diseases. Here, we report that TFF2, a novel secreted factor from OGT-deficient necroptotic hepatocytes, induced the activation of HSCs (Fig. 7*F*). We found that TFF2 promoted HSC proliferation and migration at least partially through the activation of PDGFRβ signaling. TFF2 was increased in mice with CCl_4_-induced liver injury. Given that liver injury triggers the development of HCC, these findings could potentially provide an explanation for the poor prognosis of HCC patients with a higher *Tff2* mRNA level and suggest that TFF2 may serve as a biomarker for early detection of liver injury.

The role of TFFs has not been understood in the liver until very recently. TFFs are broadly expressed in the gastrointestinal (GI) tract, liver, pancreas, kidney, and brain (29). Previous research on TFFs focused on their roles in the GI tract. Injured intestinal epithelial cells release TFFs, which promote cell proliferation and migration to restore the mucosal integrity of the GI tract (30). Interestingly, our re-examination of the RNA-sequencing data from intestinal epithelial cell–specific OGT KO mice showed the increased expression of *Tff2* transcripts (31). This result indicates that OGT may regulate *Tff2* expression in different types of cells through an unknown yet conserved mechanism. One possible mechanism is that OGT regulates Tff2 gene transcription. It has been well established that OGT can suppress gene expression through direct or indirect interaction with the promoter of its target (32). A recent study reported that O-GlcNAcylation of FOXA2 suppressed its transcriptional activity and decreased the expression of FOXA2 target genes in various liver cell lines (33). FOXA2 is predicted to bind to the promoter region of Tff2 (34). It is possible that the deletion of OGT in the liver or GI epithelial cells releases the inhibition of FOXA2 transcriptional activity and thus increases the gene expression of Tff2.

Hepatic TFF1 expression is increased in patients with hepatectomy, hepatolithiasis, cholangiocarcinoma, and HCC (35, 36, 37). The TFF3 level is commonly found in HCC patients and correlates with tumor grade (38). It has also been shown that TFF3 is involved in hepatic glucose and lipid metabolism (39, 40, 41, 42). However, the role of TFF2 is unknown in the liver. Our results reveal that TFF2, expressed and secreted by damaged hepatocytes, mediates the crosstalk between hepatocytes and HSCs. We found increased TFF2 expression in mice with CCl_4_-induced liver injury. Consistently, *Tff2* mRNA expression is also increased in a mouse model of non-alcoholic steatohepatitis fibrosis (43). A single-cell RNA-sequencing analysis of human samples revealed that *Tff2* expression is increased in hepatocytes from cirrhotic livers (44). These results suggest that TFF2 could potentially serve as a biomarker for chronic liver disease. Whether TFF2 represents a general marker for hepatic stress response or a specific modulator under certain pathological conditions is a question that remains to be answered. Future research will be needed to determine whether TFF2 could be detected in the circulation and whether neutralizing TFF2 would ameliorate liver fibrosis in different models of liver injury.

In this study, we demonstrated that the PDGFRβ signaling pathway was potentiated by TFF2 in HSCs. It has been known that AKT and ERK1/2 act downstream of PDGFRβ phosphorylation. The activation of these pathways modulates cell proliferation and migration (45). However, it is not known whether TFF2 stimulates PDGFRβ phosphorylation through direct or indirect interaction. No *bona fide* receptor has been identified for TFFs (30, 46, 47). Previous studies showed that TFF2 exerts its mitogenic effect through the CXCR4 signaling pathway in pancreatic beta cells and several other cancer cell lines (48, 49). There is also evidence that PDGFRβ functionally interacts with CXCR4 to promote cell proliferation and migration (50, 51). It is thus possible that TFF2 interacts with the CXCR4–PDGFRβ axis to promote the activation of HSCs. Future mechanistic studies are needed to elucidate the *bona fide* receptor of TFF2 in HSCs.

Previous findings have shown that O-GlcNAcylation is important for normal liver physiology, and OGT-LKO mice represent an effective animal model to investigate the cellular and molecular mechanisms underlying liver fibrosis (15, 16, 17, 18, 21). Our results suggest that the perturbation of O-GlcNAc homeostasis in hepatocytes signals to nonparenchymal cells through paracrine factors. A recent study of *in vivo* secretome profiling suggests that the secretion patterns of hepatocytes vary greatly under environmental challenges, which potentially modulates liver homeostasis (52). Intracellular O-GlcNAc levels in hepatocytes are highly sensitive to environmental stress and physiologic perturbations (32). It is likely that O-GlcNAc signaling dynamically reprograms the hepatocyte secretome that acts on other cell types to coordinate tissue responses. In the RNA-seq data obtained from OGT-LKO mice, we found that a battery of upregulated genes encode secretory proteins. We cannot exclude the possibility that secreted factors other than TFF2 also affect HSC function. Further analysis of the secretome of OGT-LKO hepatocytes would likely uncover a wealth of paracrine factors that mediate intercellular signaling in liver physiology and diseases.

## Experimental procedures

### Primary HSC isolation

The isolation of HSCs followed the protocol as described (53). Briefly, mice were perfused with a buffer containing pronase, collagenase D, and DNAse Ⅰ. Cells were digested in the digestion buffer for no longer than 20 min and filtered through a 70-μm cell strainer into a 50-ml tube. After centrifugation at 580*g* for 10 min at 4 °C, cells were washed and resuspended in Gey′s balanced salt solution B (GBSS/B) buffer. To perform gradient-mediated separation of HSCs, cells were mixed with Nycodenz solution and overlapped with 1.5 ml GBSS/B solution per 13- to 14-ml cell suspension. The cells were then centrifuged at 1380*g* for 17 min at 4 °C. After the centrifugation, HSCs were visible as a thin white layer at the interface. The cells were collected with a 5-ml pipette and resuspended in GBSS/B buffer. HSCs were centrifuged again at 580*g* for 10 min at 4 °C and then resuspended in 10-ml warm medium.

### Cell culture and conditioned medium assay

Primary hepatocytes were plated on Collagen I–coated plates (BD) in Williams’ Medium E supplemented with 10% FBS, 1 μM dexamethasone, 4 μg/ml insulin, 10 mM Hepes buffer, 2 mM L-glutamine, 8 mg/l gentamicin, antibiotic-antimycotic, and 1 mM glucose. Primary HSCs were cultured in 10% FBS Dulbecco's modified Eagle's medium (DMEM), and LX-2 cells were cultured in 2% FBS DMEM. To collect the conditioned medium from WT and OGT-LKO hepatocytes, cells were replaced with FBS-free Williams’ Medium E 4 h after seeding and cultured overnight. The medium was then collected and filtered with a 0.2-μm strainer. The medium was added to HSCs, and the cells were cultured in the conditioned medium for 16 h before harvested for further analysis. DMEM, FBS, William’s medium E, Hepes buffer, glutamine stock solution, antibiotic-antimycotic, gentamicin, and amphotericin were from Gibco. Dexamethasone, insulin, and glucose were purchased from Sigma-Aldrich.

### Silver stain of secreted proteins

The conditioned medium was harvested from the hepatocytes cultured in FBS-free William’s Medium E overnight. The medium was filtered through a 0.2-μm strainer and then applied to the centrifugal filter units Amicon 15 (Millipore) with a molecular weight cutoff of 3K. After 50-min centrifugation at 4000 rpm at 4 °C, the concentrated medium was precipitated with trichloroacetic acid (Sigma-Aldrich). The mixture was then centrifuged at 14k rpm for 5 min at 4 °C. The pellet was resuspended in 8 M urea resolubilization buffer, and proteins were quantified. The proteins were then boiled with SDS at 95 °C for 10 min. One microgram of total proteins were resolved on SDS-PAGE gels to perform the silver staining of secreted proteins. The silver staining was performed with the Pierce Silver Stain kit (Thermo Fisher) per manufacturer’s protocol. The gel was imaged with the ChemiDoc imaging system.

### Western blot

Liver tissues and cells were lysed in a buffer containing 1% Nonidet P-40, 150 mM NaCl, 0.1 mM EDTA, 50 mM Tris HCl, proteinase inhibitors, and protein phosphatase inhibitors. The lysates were then washed and boiled in SDS loading buffer. Equal amounts of protein lysates were resolved on SDS-PAGE gels and transferred to the polyvinylidene fluoride membrane. The membranes were blocked in 5% bovine serum albumin (BSA) and incubated with various primary antibodies overnight at 4 °C. After three washes, the membranes were incubated with peroxidase-conjugated secondary antibodies for 1 h and visualized with ECL chemiluminescent substrate. Antibody against p-AKT Ser 473 (4060), pan-AKT (4691), p-p44/42 MAPK (4370), p44/42 MAPK (4695), and GAPDH (2118) were purchased from Cell Signaling Technology. Antibody against PDGFRβ (ab32570) was purchased from Abcam. Antibody against αSMA (A2547) was purchased Sigma-Aldrich. Antibody against p-PDGFRβ (sc-365464) was purchased Santa Cruz. Antibodies against TFF2 (LS-C296796) and BMP8b (LS-C372895) were purchased from LSBio. Antibody against GPNMB (PA5-89716) was purchased from Invitrogen.

### RNA extraction and qPCR

Total RNA was extracted from frozen liver samples or cells with TRIzol reagent (Invitrogen). cDNA was reverse-transcribed from total RNA with Superscript III enzyme (Bio-Rad) and amplified with SYBR Green Supermix (Bio-Rad) using a LightCycler 480 real-time PCR system (Roche). All data were normalized to the expression of *36b4*. Primer sequences are listed in Table 1.

**Table 1:** Primer sequences

Name	Oligo sequence
*Tff1-F*	AGCACAAGGTGATCTGTGTCC
*Tff1-R*	GGAAGCCACAATTTATCCTCTCC
*Tff2-F*	CCTTGGTGTTTCCACCCACTT
*Tff2-R*	AGCAGCAGTTTCGACTGGC
*Tff3-F*	TTGCTGGGTCCTCTGGGATAG
*Tff3-R*	TACACTGCTCCGATGTGACAG
*Gpnmb-F*	AGAAATGGAGCTTTGTCTACGTC
*Gpnmb-R*	CTTCGAGATGGGAATGTATGCC
*Bmp8b-F*	TCCACCAACCACGCCACTAT
*Bmp8b-R*	CAGTAGGCACACAGCACACCT
*Acta2-F*	GTTCAGTGGTGCCTCTGTCA
*Acta2-R*	ACTGGGACGACATGGAAAAG
*Col1a1-F*	TAGGCCATTGTGTATGCAGC
*Col1a1-R*	ACATGTTCAGCTTTGTGGACC
*Col2a1-F*	CAGGATGCCCGAAAATTAGGG
*Col2a1-R*	ACCACGATCACCTCTGGGT
*Col3a1-F*	CTGTAACATGGAAACTGGGGAAA
*Col3a1-R*	CCATAGCTGAACTGAAAACCACC
*Mmp2-F*	GGGGTCCATTTTCTTCTTCA
*Mmp2-R*	CCAGCAAGTAGATGCTGCCT
*Timp1-F*	AGGTGGTCTCGTTGATTCGT
*Timp1-R*	GTAAGGCCTGTAGCTGTGCC
*Tgfβ-F*	CAACCCAGGTCCTTCCTAAA
*Tgfβ-R*	GGAGAGCCCTGGATACCAAC

### Immunofluorescence

Primary hepatocytes and HSCs were plated on 8-well chamber slides for immunofluorescence imaging. After indicated treatments, cells were washed three times with ice-cold PBS and fixed with 4% paraformaldehyde (PFA) for 20 min. To obtain better staining of intracellular target proteins, cells were permeabilized in 0.5% Triton X-100 for 10 min at room temperature (RT). Cells were blocked with 2% BSA in 0.5% Tween 20 in PBS and then incubated with indicated primary antibodies at 4 °C overnight in a humidified chamber. On the second day, the cells were moved from 4 °C to RT and stayed for 30 min before incubation with Alexa Fluor 488–conjugated secondary antibodies (Thermo Fisher Scientific) for 1 h in dark followed by staining with 4′,6-diamidino-2-phenylindole. The slides were mounted with VECTASHIELD antifade mounting medium (Vector Laboratories) and saved at 4 °C until imaging. For immunofluorescence of liver sections, frozen sections were moved from −80 to −20 °C for 20 min and then equilibrated at RT for another 30 min. Slides were blocked in 5% serum and 0.3% Triton PBS for 1 h in a staining box. Slides were then incubated with the primary antibody at 4 °C overnight and with the secondary antibody and 4′,6-diamidino-2-phenylindole at RT for 1 h. The slides were mounted with the fluorescence mounting medium (Dako) and stored at 4 °C.

### Cell proliferation assay

The proliferation of HSCs and LX-2 cells was determined with the Cell Proliferation kit (Millipore). HSCs were cultured in 2% FBS DMEM, and LX-2 cells were cultured in 0.5% FBS DMEM for cell proliferation assays. Cells were plated in a black-wall, clear-bottom, 96-well plate. The medium was replaced every other day with indicated treatment. Recombinant TFF2 (Novus) was dissolved in 0.1% BSA PBS, and the final concentration used was 40 nM. The plates were harvested every other day, and the assays were performed per manufacturer’s protocol. The luminescence was determined with the Tecan Infinite M200 plate reader.

### Cell migration assay

The cell migration assay was performed with the modified Boyden chamber (54). 100 μl primary HSCs suspended in 10% FBS DMEM was plated in the insert with 8-μm pores (Corning), which was placed in the well of a 24-well plate with 600-μl medium. 4 h after plating, the medium in the insert was replaced with FBS-free medium, while the medium in the well was replaced with 2% FBS DMEM with indicated treatment. The cells were incubated for 24 h. The insert was washed with PBS and fixed with 4% PFA for 2 min. The insert was then stained with 0.2% crystal violet solution (Sigma-Aldrich) for 15 min and briefly rinsed in water. Cells on the upper membrane were swabbed by a cotton tip. The insert was imaged with the ChemiDoc imaging system.

### Immunohistochemistry

Mouse livers were dissected and fixed in 4% PFA for 48 h. Immunohistochemistry was performed with the Vectastain Elite ABC HRP kit (Vector Laboratories). Paraffin-embedded sections were deparaffined, and antigen retrieval was carried out in a preheated steamer. The slides were then blocked for background peroxidase activity with hydrogen peroxide solution and unspecific binding with the blocking buffer provided in the kit. The slides were incubated with 1:50 primary antibody overnight at 4 °C and secondary antibody for 30 min at RT. The slides were counterstained with Meyer’s hematoxylin solution, dehydrated, and mounted.

### CCl_4_-induced liver injury model

CCl_4_ (Sigma-Aldrich) solution was made freshly before each injection. CCl_4_ was mixed 1:1 with olive oil (Sigma-Aldrich) and was injected at the dosage of 1 μl/g body weight. The injection was performed every other day. All procedures have been approved by the Institutional Animal Care and Use Committee of Yale University.

### Statistics

Results are presented as the mean ± SEM. The comparisons were carried out using two-tailed unpaired Student’s *t* test between two groups or one-way ANOVA followed by Tukey-adjusted multiple comparisons. Data were plotted with GraphPad Prism. A *p* value less than 0.05 was considered significant.

## Data availability

All data are contained within the article. RNA-sequencing data are available at Gene Expression Omnibus with the accession number GSE134993.

## Conflict of interest

The authors declare that they have no conflicts of interest with the contents of this article.
